# SF3B1 deficiency impairs human erythropoiesis via activation of p53 pathway: implications for understanding of ineffective erythropoiesis in MDS

**DOI:** 10.1186/s13045-018-0558-8

**Published:** 2018-02-12

**Authors:** Yumin Huang, John Hale, Yaomei Wang, Wei Li, Shijie Zhang, Jieying Zhang, Huizhi Zhao, Xinhua Guo, Jing Liu, Hongxia Yan, Karina Yazdanbakhsh, Gang Huang, Christopher D. Hillyer, Narla Mohandas, Lixiang Chen, Ling Sun, Xiuli An

**Affiliations:** 1 Department of Hematology, The First Affiliated Hospital of Zhengzhou University, Zhengzhou, 450000 Henan People’s Republic of China; 2 Laboratory of Membrane Biology, New York Blood Center, New York, NY 10065 USA; 3 Red Cell Physiology Laboratory, New York Blood Center, New York, NY 10065 USA; 4 School of Life Sciences, Zhengzhou University, Zhengzhou, Henan 450001 People’s Republic of China; 5 The State Key Laboratory of Medical Genetics and School of Life Sciences, Central South University, Changsha, 410078 People’s Republic of China; 6 Laboratory of Complement Biology, New York Blood Center, New York, NY 10065 USA; 7 Division of Experimental Hematology and Cancer Biology, Cincinnati Children’s Hospital Medical Center, Cincinnati, OH 45229 USA; 8 Department of Immunology, The Affiliated Cancer Hospital of Zhengzhou University and Henan Cancer Hospital, Zhengzhou, 450008 People’s Republic of China

**Keywords:** SF3B1, Human erythropoiesis, Apoptosis, P53

## Abstract

**Background:**

SF3B1 is a core component of splicing machinery. Mutations in SF3B1 are frequently found in myelodysplastic syndromes (MDS), particularly in patients with refractory anemia with ringed sideroblasts (RARS), characterized by isolated anemia. SF3B1 mutations have been implicated in the pathophysiology of RARS; however, the physiological function of SF3B1 in erythropoiesis remains unknown.

**Methods:**

shRNA-mediated approach was used to knockdown SF3B1 in human CD34^+^ cells. The effects of SF3B1 knockdown on human erythroid cell differentiation, cell cycle, and apoptosis were assessed by flow cytometry. RNA-seq, qRT-PCR, and western blot analyses were used to define the mechanisms of phenotypes following knockdown of SF3B1.

**Results:**

We document that SF3B1 knockdown in human CD34^+^ cells leads to increased apoptosis and cell cycle arrest of early-stage erythroid cells and generation of abnormally nucleated late-stage erythroblasts. RNA-seq analysis of SF3B1-knockdown erythroid progenitor CFU-E cells revealed altered splicing of an E3 ligase Makorin Ring Finger Protein 1 (MKRN1) and subsequent activation of p53 pathway. Importantly, ectopic expression of MKRN1 rescued SF3B1-knockdown-induced alterations. Decreased expression of genes involved in mitosis/cytokinesis pathway including polo-like kinase 1 (PLK1) was noted in SF3B1-knockdown polychromatic and orthochromatic erythroblasts comparing to control cells. Pharmacologic inhibition of PLK1 also led to generation of abnormally nucleated erythroblasts.

**Conclusions:**

These findings enabled us to identify novel roles for SF3B1 in human erythropoiesis and provided new insights into its role in regulating normal erythropoiesis. Furthermore, these findings have implications for improved understanding of ineffective erythropoiesis in MDS patients with SF3B1 mutations.

**Electronic supplementary material:**

The online version of this article (10.1186/s13045-018-0558-8) contains supplementary material, which is available to authorized users.

## Background

Erythropoiesis is an integral component of hematopoiesis. It is a process by which hematopoietic stem cells undergo multiple developmental stages to eventually generate erythrocytes. Disordered or ineffective erythropoiesis is a feature of a large number of human hematological disorders. These include Cooley’s anemia [1], congenital dyserythropoietic anemia [2], Diamond-Blackfan anemia [3], malarial anemia [4], and various bone marrow failure syndromes including myelodysplastic syndromes (MDS) [5].

Since anemia has long been recognized as a global health problem of high clinical relevance, the physiological basis for regulation of normal and disordered erythropoiesis in humans and in animals has been extensively studied. However, the primary focus of many of these studies has been on defining the roles of cytokines and transcription factors in regulating erythropoiesis. The most extensively studied regulator is erythropoietin (EPO) and its receptor (EPOR). It is firmly established that the EPO/EPOR system is essential for erythropoiesis [6–9]. At the transcriptional level, red cell development is regulated by multiple transcription factors [10], two of which, GATA1 and KLF1, are considered as master regulators of erythropoiesis [11, 12]. In addition to cytokines and transcription factors, recent studies are beginning to reveal the importance of other regulatory mechanisms such as miRNAs [13–15], histone modifiers [16], and DNA modifiers TET2 and TET3 [17] in regulating erythropoiesis.

Pre-mRNA splicing is a fundamental process in eukaryotes and is emerging as an important co-transcriptional or post-transcriptional regulatory mechanism. More than 90% of multi-exon genes undergo alternative splicing, enabling generation of multiple protein products from a single gene. In the context of erythropoiesis, one classic example is the alternative splicing of exon 16 of the gene encoding protein 4.1R. This exon is predominantly skipped in early erythroblasts but included in late-stage erythroblasts [18]. As this exon encodes part of the spectrin-actin binding domain required for optimal assembly of a mechanically competent red cell membrane skeleton [19], the importance of this splicing switch is underscored by the fact that failure to include exon 16 causes mechanically unstable red cells and aberrant elliptocytic phenotype with anemia [20]. In addition, alternative isoforms of various erythroid transcripts have been reported [21]. More recently, we documented that a dynamic alternative-splicing program regulates gene expression during terminal erythropoiesis [22]. These findings strongly imply that alternative splicing and associated regulatory factors play important roles in regulating erythropoiesis. A recent study demonstrated that knockdown of a splicing factor Mbnl1 in cultured murine fetal liver erythroid progenitors resulted in blockade of erythroid differentiation [23]. In spite of these interesting findings, the studies on the role of mRNA splicing in erythropoiesis are very limited.

RNA splicing machinery known as spliceosome carries out RNA splicing. Each spliceosome is composed of five small nuclear RNAs (U1, U2, U4, U5, U6) and a range of associated proteins [24]. Of note, recent next-generation sequencing studies have identified several mutations involving multiple components of the RNA splicing machinery, including SF3B1, SRSF2, U2AF1, ZRSR2, PRPF40B, U2AF65, and SF1 in MDS patients [25, 26]. Of these different splicing factors, SF3B1 is one of the most frequently mutated genes, and mutations in SF3B1 have been found in more than 85% of patients with refractory anemia with RARS [27, 28]. The high frequency of SF3B1 mutations in RARS makes this gene a very strong candidate responsible for the pathogenesis of this subtype of MDS. Given the fact that RARS is characterized by isolated erythroid dysplasia, we hypothesized that SF3B1 plays important roles in normal erythropoiesis by regulating the splicing of erythroid transcripts.

To define the role of SF3B1 in human erythropoiesis, we carried out detailed biological, biochemical, and gene expression analysis of erythroid cells derived from human CD34^+^ hematopoietic stem cells following knockdown of SF3B1. We show that SF3B1 knockdown led to apoptosis, cell cycle arrest, delayed erythroid differentiation, and generation of polychromatic and orthochromatic erythroblasts with abnormal nuclei. Bioinformatics and biochemical analysis revealed that increased apoptosis and cell cycle arrest is caused by activation of p53 pathway due to an isoform switch of MKRN1, a p53 E3 ligase, while impaired enucleation and generation of late-stage erythroblasts is associated with downregulation of genes involved in mitosis/cytokinesis pathway. Our findings enabled us to document critical roles for SF3B1 in regulating human erythropoiesis via previously unknown pathways. Moreover, our findings may have implications in understanding ineffective erythropoiesis in MDS patients with SF3B1 mutations.

## Methods

### Antibodies

The antibodies used for flow cytometry were as follows: mouse monoclonal antibody against human band 3 generated in our laboratory and labeled with FITC or APC as described previously [29]. Commercial antibodies used for flow cytometry were as follows: PE-CD235a (GPA), PE-CD34, FITC-CD36, and 7AAD (BD Pharmingen, USA); APC-α4 integrin (Miltenyi Biotec, USA); and PE-Cyanine7-IL-3R (CD123) and PE-Cyanine7-Annexin V (eBioscience, USA). The antibodies used for western blotting were as follows: rabbit anti-SF3B1 was from Abcam (USA); rabbit anti-MKRN1 antibody was from BETHYL (USA); rabbit anti-p53, rabbit anti-p21, rabbit anti-BBC3, and rabbit anti-BAX were from Cell Signaling (USA); monoclonal anti-actin antibody was purchased from Sigma (USA); HRP-conjugated goat anti-rabbit IgG was from Thermofisher (USA); and HRP-conjugated mouse anti-goat IgG was from Invitrogen (USA). SYTO-16 green fluorescent was from Invitrogen (USA).

CD34^+^ cell culture, fluorescence-activated cell sorting of erythroblasts, flow cytometry analysis, colony forming assay, cytospin preparation, western blotting analysis, and statistical analysis were performed as previously described [29, 30].

### MKRN1 overexpression lentiviral vector construction

Full-length MKRN1 was cloned into the modified pRRLSIN.cPPT.PGK-IRES-GFP plasmid (abbreviated as pGFP in this study) [17]. Human full-length MKRN1 was amplified by PCR using pcDNA3-Flag-MKRN1 (Addgene, catalog number: 78751) as the template. The primer sequences for the amplification are in Additional file 1: Table S1. The resultant plasmid is abbreviated as pGFP-MKRN1.

### Preparation of the lentivirus particles and shRNA-mediated knockdown in CD34^+^ cells

To prepare the lentivirus for shRNA knockdown, 293T cells were co-transfected with packaging plasmid pCMV8.9, envelope plasmid pucMDG and pLKO1 vector which expresses shRNA against luciferase or targeted gene. All of the plasmids were purchased from Sigma-Aldrich. For MRKN1 overexpression, instead of pLKO1 vector, the pGFP-MKRN1 vector expressing full-length human MKRN1 was used. Supernatant containing viral particles was collected, the viral titers were measured, and shRNA-mediated knockdown was performed as previously described [17]. The SF3B1 knockdown shRNA sequences are listed in Additional file 1: Table S1.

### Real-time quantitative RT-PCR (qRT-PCR)

The primers for SF3B1, MKRN1, TP53, CDKN1A, BAX, and BBC3 were obtained from Harvard primer bank, and the primers for the top differentially spliced transcripts were designed using Primer-BLAST. Eurofins MWG Operon LLC synthesized the primers. The sequences of all the primers are listed in Additional file 1: Table S1. The primer specificity was validated by documenting a single peak in the melt curve and further confirmed with agarose gel electrophoresis of amplified products. The real-time PCR was performed as previously described [17].

### Cell cycle analysis

EdU kit was used for cell cycle measurement according to the manufacturer’s protocol. In brief, 1 × 10^6^ cells were incubated with 10 μM EdU for 2 h at 37 °C. After incubation, the cells were harvested and washed with 3 ml of 1% BSA in PBS. Cells were then fixed, permeabilized, and stained with EdU detection cocktail as well as 7AAD. The staining of EdU and 7AAD was analyzed by flow cytometry. Data were collected and analyzed using FlowJ, and the data are expressed as EdU fluorescence intensity versus 7AAD.

### Ectopic expression of MKRN1 in SF3B1 knockdown cells

Lentivirus particles of MKRN1 expression construct were packaged as described above. SF3B1 shRNA lentivirus particles and pGFP or pGFP-MKRN1 overexpression lentivirus particles were co-transfected into CD34^+^ cells on day 2 of culture. Puromycin was added for selection of SF3B1 shRNA transduced cells. GFP^+^ cells (co-transduced with SF3B1-shRNA2/pGFP or SF3B1-shRNA2/pGFP-MKRN1) were sorted on day 7 by FACS. The sorted cells were cultured under erythroid differentiation condition.

### Treatment of erythroblasts with polo-like kinase-1 (PLK1) inhibitor

CD34^+^ cells were cultured as previously described. On day 12, cells were treated with DMSO or PLK1 inhibitor (Selleckchem, USA; catalog no. S7720) dissolved in DMSO at the final concentration of 100 μM. Cytospins were prepared as described above.

### RNA-seq analysis and bioinformatic analysis

RNA was extracted from stage-matched luciferase control and SF3B1 knock down erythroid cells. cDNA Libraries were prepared using the standard illumina TruSeq kit and sequenced at Beijing Genomics Institute (BGI, China) using the Illumina HiSeq 4000 platform. Sequencing effort produced ~ 130 million paired-end 100 bp reads per sample. Quality control was performed on the sequenced reads, and low-quality reads were removed. The reads were then aligned with Tophat2 [31] short read aligner following the protocol detailed in Trapnell et al. [32]. Splice aware alignment was performed using cufflinks to assemble transcripts with the human hg19 genome as reference. Gene expression analysis was performed pair-wise between the luciferase and SF3B1 shRNA knockdown at each development stage using cuffdiff. Gene set enrichment analysis was performed using the pre-ranked list analysis against the curated gene sets of canonical pathways [33, 34]. Differentially expressed genes were extracted using the cummerbund R package. Differentially spliced genes where annotated and extracted using spliceR [35].

## Results

### Expression of SF3B1 during human erythroid differentiation

To explore the role of SF3B1 in human erythropoiesis, we first examined the expression of SF3B1 in highly purified erythroid cells at each distinct developmental stage derived from cord blood CD34^+^ cell cultures. SF3B1 is abundantly expressed at all stages of erythroid differentiation as assessed by RNA-seq analysis of SF3B1 transcript expression (Additional file 2: Figure S1). This pattern of expression of SF3B1 was further confirmed by real-time PCR (Additional file 2: Figure S1). Western blot analysis and analysis of proteomic data of terminally differentiated erythroblasts [36] showed that SF3B1 protein levels decreased in late-stage erythroblasts (Additional file 2: Figure S1).

### Knockdown of SF3B1 severely impairs erythroid progenitor growth

**Fig. 1:**
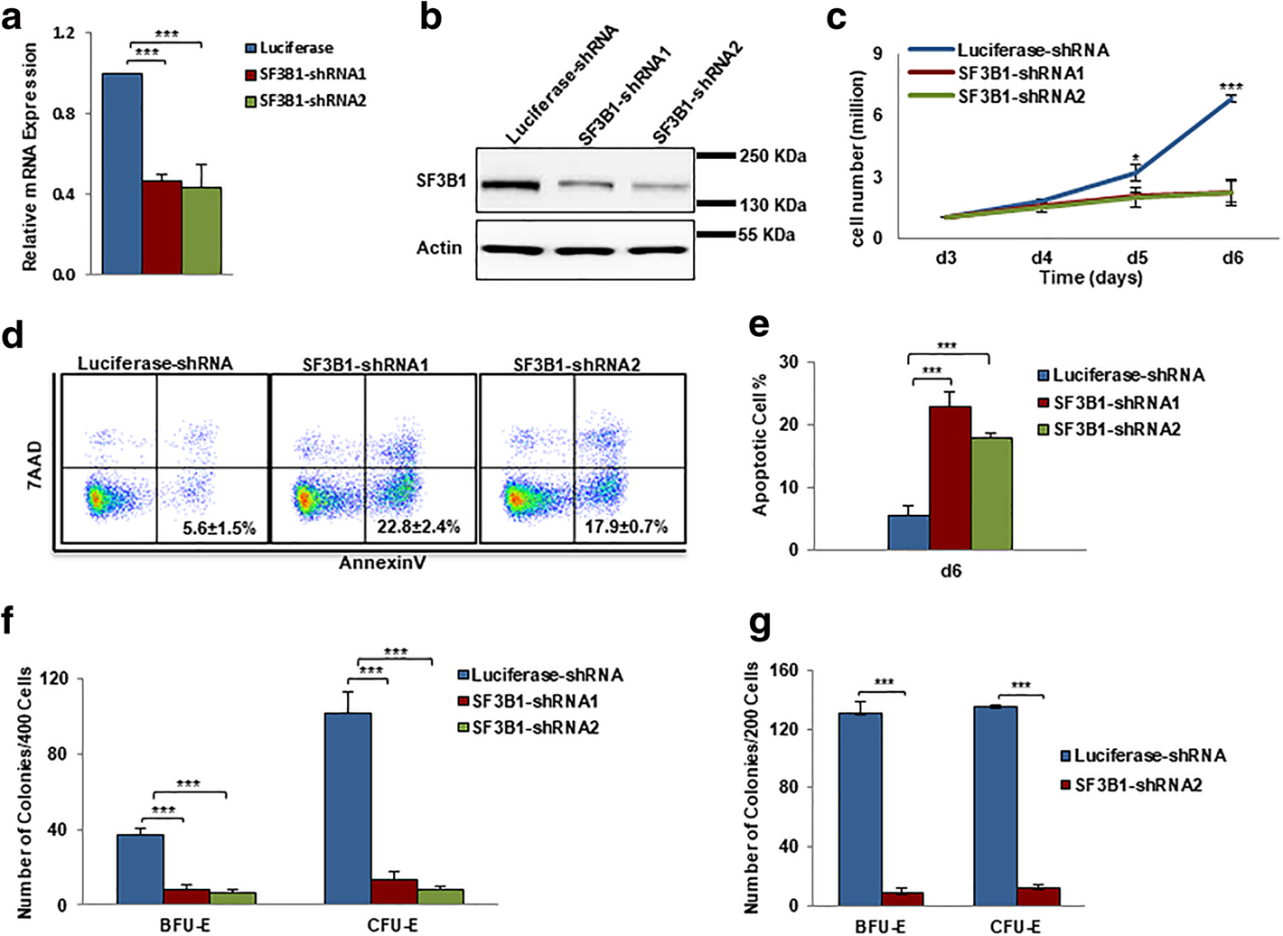
Effects of SF3B1 knockdown on proliferation of erythroid progenitors. **a** qRT-PCR results showing SF3B1 mRNA expression levels in erythroblasts transduced with lentivirus containing luciferase-shRNA or SF3B1-shRNA, and cultured for 6 days. β-actin was used as internal calculator. Bar plot represents mean ± SD of triplicate samples. **b** Representative western blotting showing SF3B1 protein levels in erythroblasts transduced with lentivirus containing luciferase-shRNA or SF3B1-shRNA, and cultured for 6 days. **c** Growth curves of cells transduced with lentivirus containing luciferase-shRNA or SF3B1-shRNA. **d** Representative flow cytometry profiles of apoptosis as assessed by dual staining of Annexin V and 7AAD at day 6 of culture. **e** Quantitative analysis of apoptosis from three independent experiments. **f** Colony-forming ability of cells cultured for 6 days, which contain mixed populations of cells that include BFU-E cells, CFU-E cells, and proerythroblasts. **g** Colony-forming ability of sorted BFU-E and CFU-E cells using IL-3R, CD34, and CD36 as surface markers [30]. ***P < 0.001

Erythropoiesis is a complex process that can be divided into early-stage erythropoiesis and terminal erythroid differentiation. During early-stage erythropoiesis, HSC cells differentiate to generate the erythroid progenitors: BFU-E (burst-forming-unit-erythroid) cells that further differentiate to generate CFU-E (colony-forming-unit-erythroid) cells. We examined the effects of SF3B1 knockdown on early-stage erythropoiesis that occurs mainly from day 4 to day 7 of culture under our erythroid differentiation condition. On day 6 of culture, > 50% knockdown efficiency was achieved in erythroid cells by two independent shRNAs at the mRNA level (Fig. 1a) and at protein level (Fig. 1b). Importantly, SF3B1 knockdown severely inhibited erythroid cell growth (Fig. 1c). To examine the cause for this impaired cell growth, we measured the extent of cell apoptosis. The representative profiles of dual staining with 7AAD and Annexin V of cells at day 6 of culture are shown in Fig. 1d. Quantitative analysis reveals that the percentage of Annexin V^+^ cells was increased from ~ 5% for luciferase shRNA-transduced cells to ~ 20% for SF3B1 shRNA-transduced cells (Fig. 1e). The impaired growth of SF3B1 shRNA-transduced erythroid progenitors was further validated by their failure to give rise to BFU-E and CFU-E colonies compared to luciferase shRNA-transduced cells (Fig. 1f). To further confirm these findings, we performed colony-forming assays using cells sorted based on cell surface markers that define BFU-E and CFU-E stages. As shown in Fig. 1g, the sorted BFU-E and CFU-E cells also failed to give rise to erythroid colonies as a consequence of increased apoptosis. Taken together, these findings imply that SF3B1 knockdown impairs proliferation of erythroid progenitors.

### SF3B1 knockdown impairs terminal erythroid differentiation

**Fig. 2:**
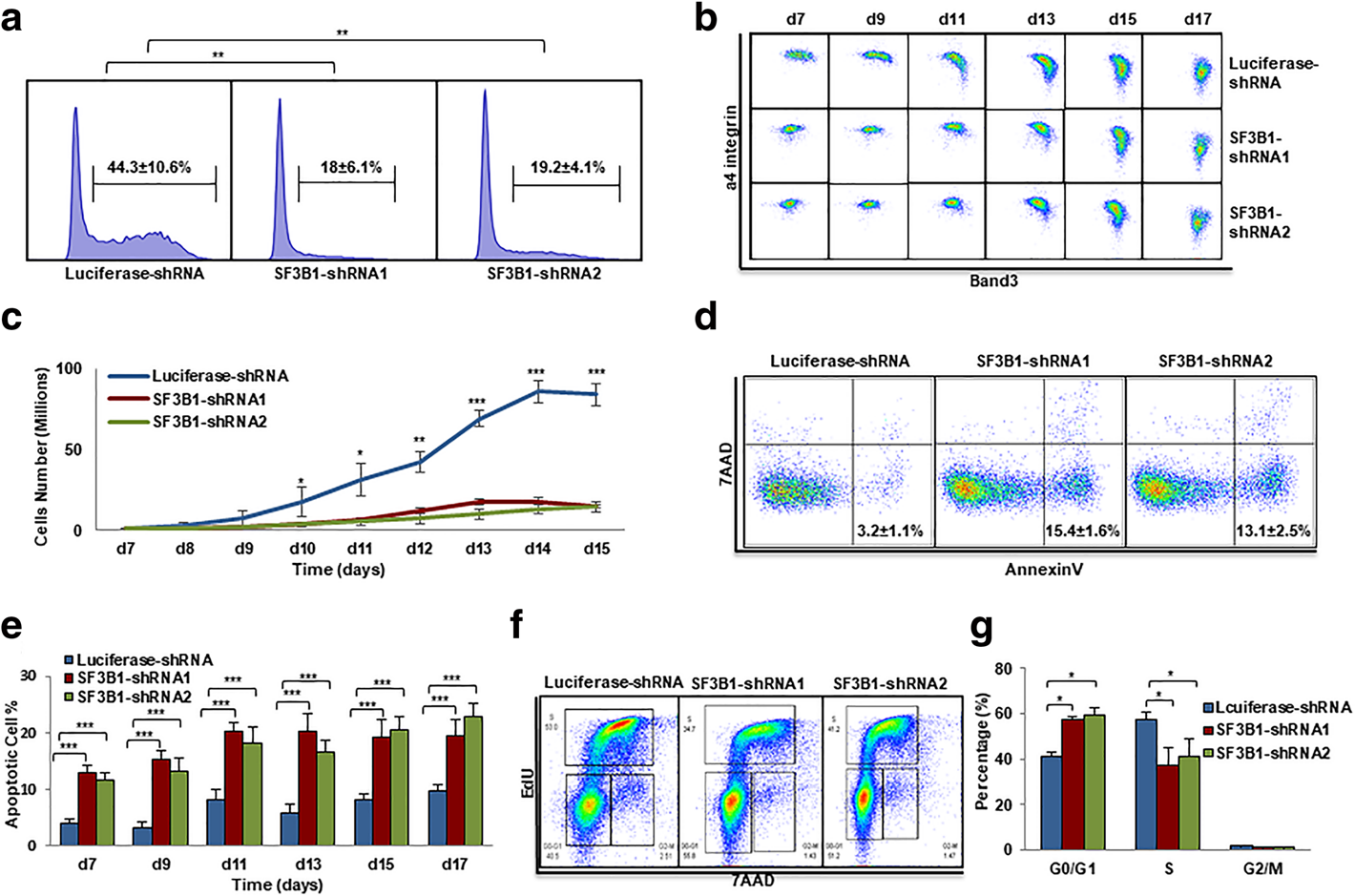
Effects of SF3B1 knockdown on terminal erythroid differentiation. **a** Flow cytometry analysis showing the percentage of GPA-positive cells on day 7. **b** Flow cytometry analysis showing the expression of α4 integrin and band 3 of erythroid cells cultured for different days as indicated. **c** Growth curves of cells transduced with lentivirus containing luciferase-shRNA or SF3B1-shRNA. **d** Representative flow cytometry profiles of apoptosis as assessed by dual staining of Annexin V and 7AAD of cells cultured for 9 days. **e** Quantitative analysis of apoptosis from three independent experiments. **f** Representative flow cytometry profiles of cell cycle as assessed by EdU and 7AAD staining of cells cultured for 7 days. **g** Quantitative analysis of cell cycle from three independent experiments. *P < 0.05, **P < 0.01, ***P < 0.001

We then examined the effects of SF3B1 knockdown on terminal erythroid differentiation, the process during which morphologically recognizable proerythroblasts derived from CFU-E cells undergo 4 to 5 mitosis to generate enucleated reticulocytes. The differentiation of CFU-E cells to proerythroblasts is characterized by the surface expression of the erythroid specific marker glycophorin A (GPA). As shown in Fig. 2a, while more than 40% of luciferase shRNA-transduced cells were GPA positive on day 7, less than 20% of SF3B1 shRNA-transduced cells were GPA positive, implying SF3B1 knockdown impairs differentiation of CFU-E to proerythroblasts. Differentiation of proerythroblasts to late-stage erythroblasts was monitored by flow cytometry using α4 integrin and band 3 as surface markers. SF3B1 knockdown delayed terminal erythroid differentiation that occurs from day 9 to day 13 in the culture system as reflected by the impaired downregulation of α4 integrin and delayed upregulation of band 3 (Fig. 2b). However, the expression patterns of α4 integrin and band 3 was similar between luciferase-shRNA-transduced and SF3B1-shRNA-transduced cells on days 15 and 17 of culture implying the eventual effective completion of terminal erythroid differentiation of surviving cells (Fig. 2b).

We also monitored cell growth in our erythroid culture system from day 7 to 14. While cells transduced with luciferase-shRNA proliferated exponentially from day 7 to day 14 with cell numbers increasing from 1 million to ~ 90 million (Fig. 2c), the rate of growth was significantly impaired for cells transduced with SF3B1-shRNAs with cell numbers increasing from 1 million to only ~ 15 million. To define the mechanistic basis for the impaired cell growth, we measured the extent of cell apoptosis. The representative profiles of dual staining with 7AAD and Annexin V of cells at day 9 of culture are shown in Fig. 2d. The Annexin V^+^ population increased from ~ 3% for luciferase-shRNA-transduced cells to ~ 15% for SF3B1-shRNA-transduced cells. Quantitative analysis of apoptosis data from three independent experiments is shown in Fig. 2e. We also monitored changes in cell cycle by EdU incorporation assay, and the representative profiles of the cell cycle analysis are shown in Fig. 2f. Quantitative analysis of data from three independent experiments showed an increased G1 phase in conjunction with decreased S phase following SF3B1 knockdown (Fig. 2g). These findings demonstrate that the severely impaired growth of erythroblasts following knockdown of SF3B1 is a consequence of increased apoptosis and cell cycle arrest.

### Generation of erythroblasts with abnormal nucleus following SF3B1 knockdown

**Fig. 3:**
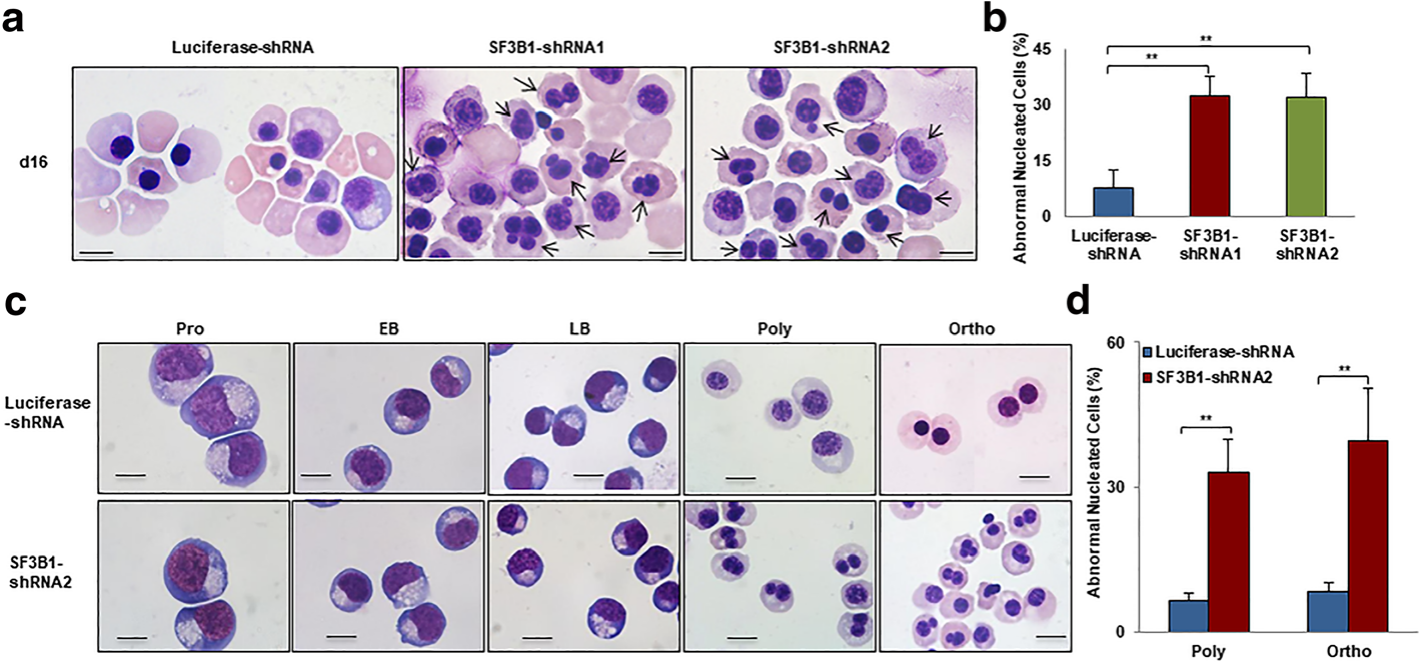
Generation of erythroblasts with abnormal nucleus upon SF3B1 knockdown. **a** Representative cytospin images of erythroblasts cultured for 16 days. **b** Quantitative analysis of abnormally nucleated erythroblasts cultured for 16 days from three independent experiments. **c** Representative cytospin images of sorted erythroblasts at each distinct developmental stage. **d** Quantitative analysis of abnormally nucleated polychromatic and orthochromatic erythroblasts from three independent experiments. Scale bar 10 μm. *P < 0.05, **P < 0.01

When examining the effects of SF3B1 knockdown on morphology of erythroid cells, we surprisingly found that many terminally differentiated erythroblasts exhibited abnormal nuclei following SF3B1 knockdown (Fig. 3a). Quantitative analysis of data derived from three independent experiments showed that while < 10% of control late-stage erythroblasts had abnormal nuclear morphology, knockdown of SF3B1 increased the percentage of cells with abnormal nucleus to ~ 30% (Fig. 3b). To determine the developmental stage at which this abnormal nuclear morphology occurs, we sorted erythroblasts at distinct developmental stages. As shown in Fig. 3c, the generation of abnormal nucleus occurred only at polychromatic and orthochromatic erythroblast stages but not at earlier stages of development. The percentage of cells with abnormal nucleus was ~ 30% for polychromatic erythroblasts and ~ 40% for orthochromatic erythroblasts (Fig. 3d).

### Activation of p53 pathway in SF3B1-knockdown CFU-E cells

**Fig. 4:**
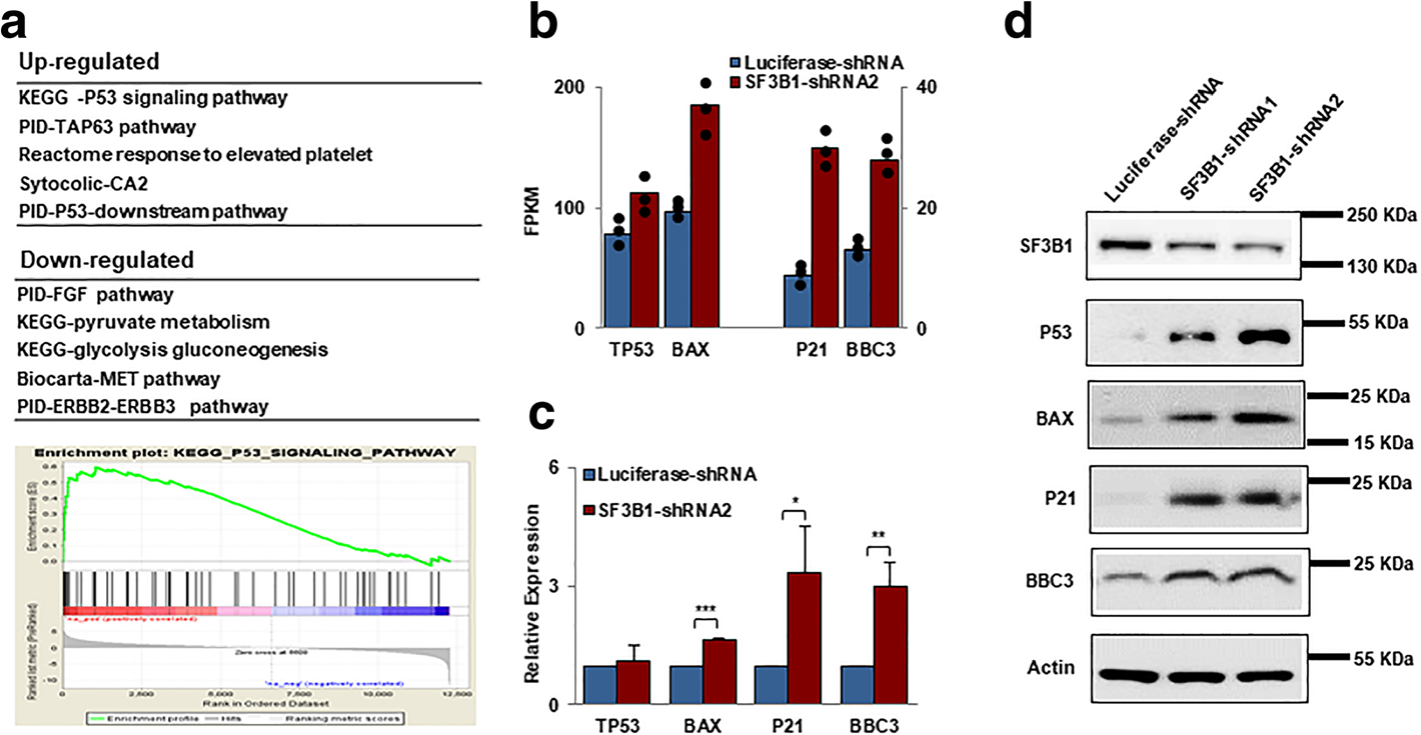
Activation of p53 pathway in SF3B1-knockdown CFU-E cells. **a** The top 5 up- and downregulated pathways revealed by GSEA analysis of the differentially expressed genes between luciferase and SF3B1 knockdown CFU-E cells. **b** The mRNA levels of TP53, BAX, p21, and BBC3 revealed by RNA-seq analysis. **c** The mRNA levels of TP53, BAX, p21, and BBC3 revealed by real-time PCR analysis; β-actin was used as internal control. *P < 0.05, **P < 0.01, ***P < 0.01. **d** Representative western blotting showing the expression of P53, BAX, p21, and BBC3 in erythroblasts cultured for 7 days

To delineate the underlying molecular mechanisms for the observed changes in apoptosis and cell cycle following SF3B1 knockdown, we performed RNA-seq analysis on purified luciferase-knockdown and SF3B1-knockdown CFU-E cells. Bioinformatics analysis of the RNA-seq data revealed that 255 genes were differentially expressed, of which 167 genes were downregulated and 88 genes were upregulated. List of the differentially expressed genes is shown in Additional file 3: Table S2. To define the pathways affected by SF3B1 knockdown, we performed gene set enrichment analysis. Figure 4a shows the top five upregulated and the top five downregulated pathways. Notably, consistent with changes in apoptosis and cell cycle arrest, the highest upregulated pathway is the p53 pathway. As the p53-induced cell cycle arrest and apoptosis are mediated by cell cycle inhibitor p21 [37, 38] and by transcriptional activation of Bcl-2 family pro-apoptotic genes such as Bax and BBC3 (PUMA) respectively [39–41], we compared the levels of expression of p53 and its downstream targets between control and SF3B1 knockdown cells. The RNA-seq data revealed that while there is no difference in p53 mRNA levels, the mRNA levels of Bax, p21, and BBC3 are increased in SF3B1-knockdown CFU-E cells (Fig. 4b). These mRNA expression levels were further confirmed by real-time PCR (Fig. 4c). Importantly, western blot analysis documented the increased protein levels of downstream targets of p53, p21, BAX, and BBC3, but surprisingly, p53 protein level was also increased in spite of lack of increase in its mRNA levels (Fig. 4d).

### Effects of SF3B1 knockdown on RNA splicing

**Fig. 5:**
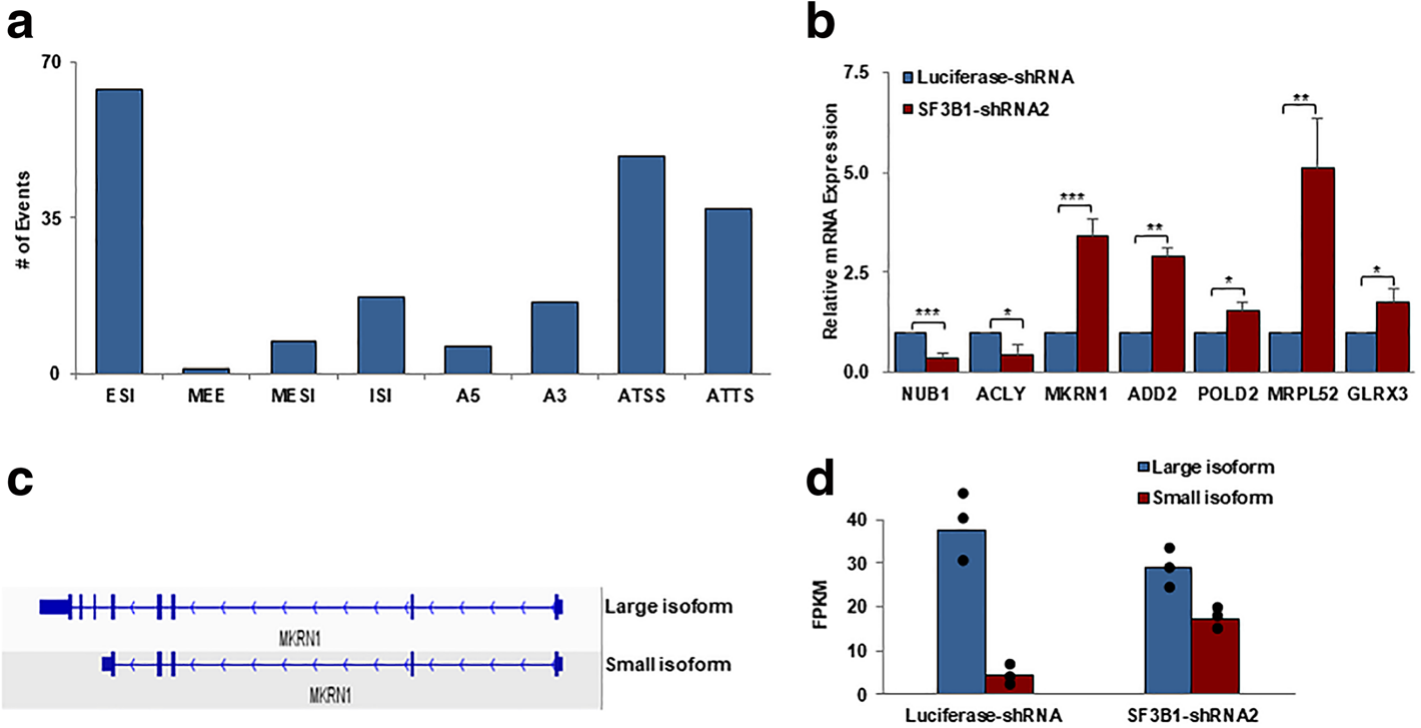
Effects of SF3B1 knockdown on splicing in CFU-E cells. **a** The most frequent splicing events by type after SF3B1 knockdown: ESI, exon skipping/inclusion; MESI, multiple exon skipping/inclusion; ISI, intron skipping/inclusion; A5, alternative 5′ splice site; A3, alternative 3′ splice site; ATSS, alternative transcription start site; ATTS, alternative transcription termination site; MEE, mutually exclusive exons. **b** Expression of differentially spliced transcripts checked by real-time PCR. **c** Schematic expression of two MKRN1 transcripts. **d** Bar graphs showing the large and small isoforms of MKRN1 in luciferase control groups and SF3B1 knockdown groups by the analysis of RNA-seq data. Dot plots represent 3 independent experiments

As SF3B1 is the core component of the splicesome and is involved in the 3′ splice site recognition [42, 43], we analyzed the effects of SF3B1 knockdown on RNA splicing. We found that 288 transcripts were differentially spliced following SF3B1 knockdown in CFU-E cells. The list of differentially spliced transcripts is shown in Additional file 4: Table S3. Of the 288 differentially expressed transcripts, 198 could be categorized into different types of splicing events. The schematic presentation of types of altered splicing events is shown in Additional file 2: Figure S2. As shown in Fig. 5a, the most frequent splicing events noted were exon skipping/inclusion (ESI), alternative transcription start site (ATSS), and alternative transcription termination site (ATTS). We confirmed the expression of these differentially spliced transcripts by real-time RT-PCR (Fig. 5b). Amongst the differentially spliced genes, we focused our attention on Makorin Ring Finger Protein 1 (MKRN1) since it has been previously reported that the MKRN1 E3 ligase controls cell cycle arrest and apoptosis by regulating p53 and p21 [44]. As shown in Fig. 5c, there are two MKRN1 transcripts, which we termed large and small isoforms. While only the large isoform is predominantly expressed in control cells, SF3B1 knockdown resulted in decreased expression of the large isoform accompanied by increased expression of the smaller isoform (Fig. 5d).

### Activation of p53 pathway is due to decreased expression of large MKRN1 isoform

**Fig. 6:**
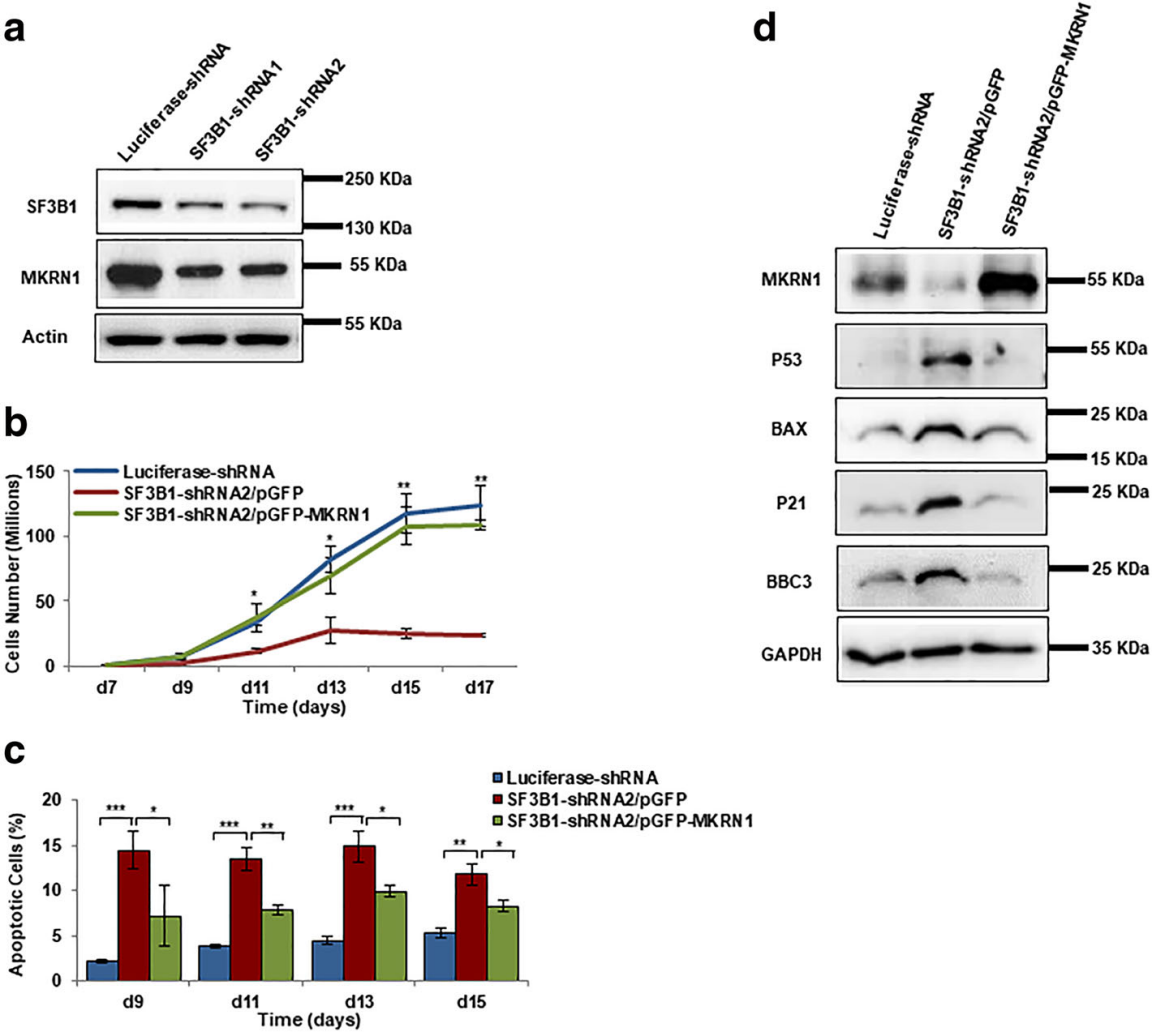
Activation of p53 pathway is due to decreased expression of large isoform of MKRN1. **a** Representative western blotting showing the expression of SF3B1 and MKRN1 large isoform in cells transduced with luciferase-shRNA and SF3B1-shRNA. **b** Growth curves of cells transduced with luciferase-shRNA, SF3B1-shRNA2/pGFP, and SF3B1-shRNA2/pGFP-MKRN1. **c** Quantitative analysis of apoptosis of cells transduced with luciferase-shRNA, SF3B1-shRNA2/pGFP, and SF3B1-shRNA2/pGFP-MKRN1. **d** Representative western blot analysis showing the expression of p53, BAX, p21, and BBC3 in cells transduced with luciferase-shRNA, SF3B1-shRNA2/pGFP, and SF3B1-shRNA2/pGFP-MKRN1

The finding that the increased p53 protein level is not accompanied by increased mRNA level suggested that the increased levels of p53 protein could be due to impaired protein degradation. It has been well documented that p53 degradation is mediated by MDM2, the first identified E3 ubiquitin ligase for p53 [45, 46]. More recently, MKRN1 has also been identified as an E3 ligase that can target p53 for degradation [44]. Our finding that SF3B1 knockdown leads to isoform switch of MKRN1 strongly suggests a potential link between SF3B1 knockdown, MKRN1 splicing change, and p53 activation. To test this thesis, we first examined the protein levels of MKRN1. As shown in Fig. 6a, the large isoform of MKRN1 is significantly decreased in cells following SF3B1 knockdown. To provide further evidence that SF3B1 knockdown-induced changes are due to decreased expression of large isoform of MKRN, we ectopically expressed the large isoform of MKRN1in SF3B1-knockdown cells. As shown in Fig. 6b, transfection with GFP-MKRN1 but not GFP alone rescued the cell growth. Figure 6c shows that the apoptotic phenotype of SF3B1-knockdown cells were also rescued following ectopic expression of the large isoform of MKRN1. Moreover, as shown in Fig. 6d, ectopic expression of GFP-MKRN1 large isoform also rescued the protein levels of p53, p21, Bax, and BBC3.

### Effects of SF3B1 knockdown on gene expression of polychromatic and orthochromatic erythroblasts

**Fig. 7:**
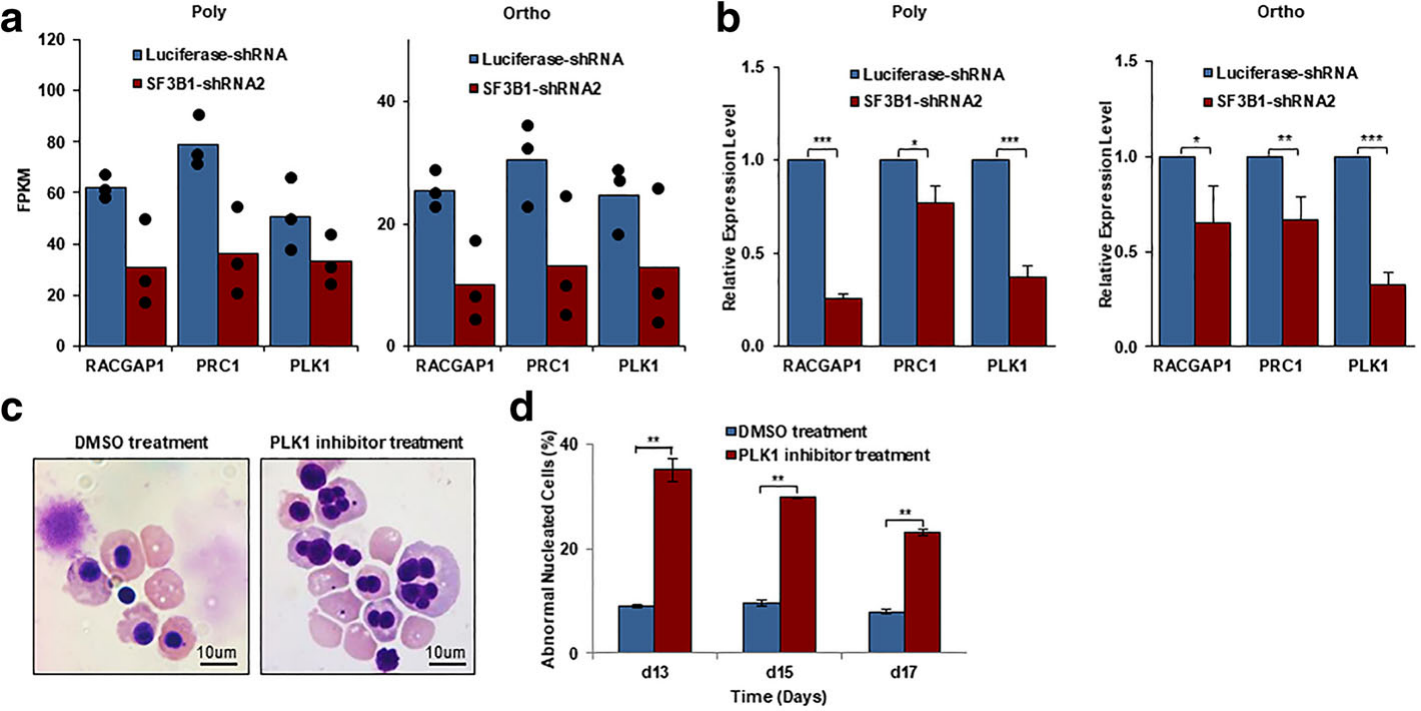
Downregulation of mitosis/cytokinesis pathway in polychromatic and orthochromatic erythroblasts. **a** RNA-seq expression data of RACGAP1, PRC1, and PLK1 in luciferase and SF3B1 knockdown polychromatic and orthochromatic erythroblasts. Dot plots represent 3 independent experiments. **b** mRNA expression levels of RACGAP1, PRC1, and PLK1 as assessed by real-time PCR using β-actin as internal calibrator. **c** Representative images of morphology from DMSO and PLK1 inhibitor treated cells. **d** Quantification of abnormally nucleated erythroblasts from three independent experiments. **P < 0.01

In addition to apoptosis and cell cycle arrest, another phenotypic change noted during terminal erythroid differentiation is the generation of erythroblasts with abnormal nuclei at the polychromatic and orthochromatic erythroblast stages. To explore the molecular basis for these changes, we performed RNA-seq analysis on luciferase-knockdown and SF3B1-knockdown polychromatic and orthochromatic erythroblasts. Bioinformatics analysis revealed that 602 genes were differentially expressed between luciferase-knockdown and SF3B1-knockdown polychromatic erythroblasts and 461 genes are differentially expressed between luciferase-knockdown and SF3B1-knockdown orthochromatic erythroblasts. The list of these differentially expressed genes is shown in Additional file 3: Table S2. Gene set enrichment analysis revealed that the top five downregulated pathways are associated with mitosis/cytokinesis pathway and DNA replication pathway (Additional file 5: Table S4). As abnormal nucleus is most likely to be associated with defects in mitosis/cytokinesis, we focused our attention on this group of genes and found that several mitosis/cytokinesis genes such as the PRC1, PLK1, and RACGAP1 are indeed the top downregulated genes in both polychromatic and orthochromatic erythroblasts. Decreased mRNA levels of PRC1, PLK1, and RACGAP1 following SF3B1 knockdown were noted by RNA-seq analysis (Fig. 7a) and validated by real-time PCR (Fig. 7b). Importantly, treatment of late-stage erythroblasts by PLK1 inhibitor also resulted in the generation of abnormally nucleated erythroblasts (Fig. 7c). Quantitative analysis reveals that while < 10% of polychromatic and orthochromatic erythroblasts have abnormal nucleus in control group, PLK1 treatment increased the percentage of polychromatic/orthochromatic erythroblasts with abnormal nucleus to ~ 30% (Fig. 7d).

## Discussion

It has been shown that MDS patients with SF3B1 mutation are characterized by isolated anemia. However, it is not clear whether deficiency of SF3B1 directly contributes to the anemia of the patients. Our finding that SF3B1 knockdown significantly impairs human erythropoiesis demonstrates the critical role of SF3B1 in normal human erythropoiesis and implies the contribution of SF3B1deficiency to the anemia of MDS patients with SF3B1 mutation. Moreover, we have identified pathways by which SF3B1 regulates erythropoiesis. Our findings provide new and novel insights into erythroid cell development and may have implications in understanding ineffective erythropoiesis in MDS patients with SF3B1 mutations.

Erythropoiesis is a complex process involving multiple developmental stages. Establishment of methods for isolation of human erythroid cells at distinct developmental stages enables the study of stage-specific changes in erythropoiesis in various human disorders [29, 30, 47]. We have recently documented that TET3 knockdown impaired human terminal erythroid differentiation without affecting erythroid progenitors [17]. In the present study, we show that SF3B1 knockdown had effects on both erythroid progenitors and on terminal erythroid differentiation. For the development of effective treatment strategies for treatment of anemia due to disordered erythropoiesis, it will be important to identify stage-specific defects in erythroid differentiation in various disorders. For example, as erythropoietin stimulates proliferation of erythroid progenitors, patients whose anemia is characterized by defects in late-stage erythropoiesis are resistant to erythropoietin therapy. On the other hand, recent studies have shown that an activin receptor IIA ligand trap corrects anemia by promoting late-stage erythropoiesis [48, 49].

Since the discovery of SF3B1 mutation in MDS 7 years ago [25], studies of SF3B1 function have been an active area of investigation. However, most studies to date have primarily focused either on the analysis of SF3B1 mutations in various diseases [50, 51], or on the effects of SF3B1 mutations on RNA splicing [52, 53]. In contrast, there are very few studies regarding the effects of SF3B1 deficiency on cellular function. Our finding that SF3B1 knockdown significantly impaired the growth of primary erythroid cells is consistent with a previous report on the effect of SF3B1 knockdown on the growth of several myeloid cell lines [52]. These findings demonstrate a critical role for SF3B1 in cell growth. The significantly impaired growth of SF3B1-knockdown erythroid cells strongly suggests that deficiency of SF3B1 can lead to anemia. However, haploinsufficiency of Sf3b1 is not associated with anemia in mice [54, 55]. One possible explanation for the lack of erythropoietic defects in Sf3b1heterozygous mice could be due to the fact that haploinsufficiency does not manifest an erythroid phenotype in mice similar to the reported for RPS19 for Diamond-Blackfan anemia [56] and for Sec23B for congenital dyserythropoietic anemia type II [57].

RARS is characterized by the presence of more than 15% of ring sideroblasts. Our data revealed that knockdown of SF3B1 led to a slight but not statistically significant increase in the percentage of ring sideroblasts (5% for SF3B1 knockdown versus 3% for luciferase control). Ring sideroblasts are red cell precursors with mitochondrial iron accumulation around the nucleus. Most of the iron deposited in perinuclear mitochondria of ring sideroblasts is present in the form of mitochondrial ferritin [58]. It is interesting to note that the expression of mitochondria ferritin is very low in normal erythroid precursors and its expression is significantly increased in sideroblasts [59]. Therefore, one possible explanation for the difficulty in reproducing ring sideroblasts in vitro is likely to be the consequence of lack of mitochondria ferritin expression in our in vitro erythroid culture system.

In exploring the molecular basis for the observed phenotypic changes, we performed RNA-seq analysis. Bioinformatics analysis of our RNA-seq data revealed significant change in many pathways. Our findings appear to be inconsistent with findings from the study by Visconte et al. who performed RNA-seq analysis on unfractionated bone marrow cells from Sf3b1^+/+^ and Sf3b1^+/−^ mice and noted that global differential expression analysis did not find any significant difference showed between the two groups [60]. We would like to point out that our RNA-seq was performed specifically on erythroid cells. These findings suggest the relative selective effects of SF3B1 deficiency on gene expression in erythroid cells.

One striking finding of our study is that SF3B1 knockdown led to activation of p53 pathway and this activation is due to altered splicing of MKRN1, an E3 ligase that targets p53 for degradation [44]. The link between SF3B1, MKRN1, and p53 pathway is further supported by the findings that ectopic expression of the large isoform MKRN1 in SF3B1 knockdown erythroid cells rescued cell growth and restored the expression levels of p53 and p53 downstream targets. Activation of p53 has been implicated in impaired erythropoiesis of DBA in zebrafish, mouse, and human model systems [61–63]. Our findings not only confirm the previous findings that activation of p53 impairs erythropoiesis but also identified a previously unknown mechanism by which p53 activation is regulated in erythroid cells.

In addition to increased apoptosis and cell cycle arrest, SF3B1 knockdown also led to generation of late-stage erythroblasts with abnormal nuclei. Notably, the abnormal nuclei were only observed in SF3B1 knockdown-polychromatic and orthochromatic erythroblasts. This may be due to the selective upregulation of several cytokinesis-/mitosis-associated genes at polychromatic and orthochromatic stages, which are downregulated by SF3B1 knockdown.

The effects of SF3B1 mutations on splicing have been extensively studied [52, 53, 64]. These studies documented that SF3B1 mutations lead to altered splicing by promoting the usage of cryptic 3′ splicing site [42, 43]. In contrast, we documented that the most common changes in splicing pattern due to quantitative deficiency of SF3B1 are exon skipping/inclusion (ESI), alternative transcription start site (ATSS), and alternative transcription termination site (ATTS). Our findings further support the current concept that SF3B1 mutation is a gain-of-function rather than loss-of-function.

## Conclusions

In summary, our findings demonstrate that SF3B1 plays important roles in human erythropoiesis and it does so by maintaining the proper splicing program during erythropoiesis. In particular, we documented the functional consequence of alternative splicing of MKRN1. The functional sequela of splicing changes in other genes due to SF3B1 knockdown is worthy of further investigation.

## Additional files

Additional file 1:Table S1. Primers and shRNA sequences. (XLSX 11 kb)

Additional file 2: Figure S1. Expression of SF3B1 during human erythroid differentiation. **Figure S2.** A schematic structure of each alternative splicing type, along with the associated names and abbreviations. (ZIP 4064 kb)

Additional file 3: Table S2. Differentially expressed genes between stage-matched luciferase control and SF3B1 knockdown erythroid cells. (XLSX 239 kb)

Additional file 4: Table S3. Differentially spliced genes between stage-matched luciferase control and SF3B1 knockdown erythroid cells. (XLSX 169 kb)

Additional file 5: Table S4. Upregulated and downregulated pathways of SF3B1 knockdown. (XLSX 30 kb)

## Abbreviations

ATSS: Alternative transcription start site
ATTS: Alternative transcription termination site
BFU-E: Burst-forming-unit-erythroid
CFU-E: Colony-forming-unit-erythroid
EPO: Erythropoietin
EPOR: Erythropoietin receptor
ESI: Exon skipping/inclusion
MDS: Myelodysplastic syndromes
MKRN1: Makorin Ring Finger Protein 1
PLK1: Polo-like kinase 1
RARS: Refractory anemia with ringed sideroblasts

## References

[1] Ginzburg Y, Rivella S (2011). β-thalassemia: a model for elucidating the dynamic regulation of ineffective erythropoiesis and iron metabolism. Blood.

[2] Wickramasinghe SN, Wood WG (2005). Advances in the understanding of the congenital dyserythropoietic anaemias. Br J Haematol.

[3] Lipton JM, Ellis SR (2009). Diamond-Blackfan anemia: diagnosis, treatment, and molecular pathogenesis. Hematol Oncol Clin North Am.

[4] Haldar K, Mohandas N (2009). Malaria, erythrocytic infection, and anemia. Hematol Am Soc Hematol Educ Progr.

[5] Ebert BL, Galili N, Tamayo P, Bosco J, Mak R, Pretz J, et al. An erythroid differentiation signature predicts response to lenalidomide in myelodysplastic syndrome. Appelbaum FR, editor. PLoS Med. 2008;5:e35. 10.1371/journal.pmed.0050035 PMC2235894 18271621

[6] Lin FK, Suggs S, Lin CH, Browne JK, Smalling R, Egrie JC (1985). Cloning and expression of the human erythropoietin gene. Proc Natl Acad Sci U S A.

[7] D’Andrea AD, Lodish HF, Wong GG (1989). Expression cloning of the murine erythropoietin receptor. Cell.

[8] Wu H, Liu X, Jaenisch R, Lodish HF (1995). Generation of committed erythroid BFU-E and CFU-E progenitors does not require erythropoietin or the erythropoietin receptor. Cell.

[9] Lin CS, Lim SK, D’Agati V, Costantini F (1996). Differential effects of an erythropoietin receptor gene disruption on primitive and definitive erythropoiesis. Genes Dev.

[10] Cantor AB, Orkin SH (2002). Transcriptional regulation of erythropoiesis: an affair involving multiple partners. Oncogene.

[11] Siatecka M, Bieker JJ (2011). The multifunctional role of EKLF/KLF1 during erythropoiesis. Blood.

[12] Pevny L, Lin CS, D’Agati V, Simon MC, Orkin SH, Costantini F (1995). Development of hematopoietic cells lacking transcription factor GATA-1. Development.

[13] Dore LC, Amigo JD, Dos Santos CO, Zhang Z, Gai X, Tobias JW (2008). A GATA-1-regulated microRNA locus essential for erythropoiesis. Proc Natl Acad Sci U S A.

[14] Zhu Y, Wang D, Wang F, Li T, Dong L, Liu H (2013). A comprehensive analysis of GATA-1-regulated miRNAs reveals miR-23a to be a positive modulator of erythropoiesis. Nucleic Acids Res.

[15] Zhao G, Yu D, Weiss MJ (2010). MicroRNAs in erythropoiesis. Curr Opin Hematol.

[16] Ji P, Yeh V, Ramirez T, Murata-Hori M, Lodish HF (2010). Histone deacetylase 2 is required for chromatin condensation and subsequent enucleation of cultured mouse fetal erythroblasts. Haematologica.

[17] Yan H, Wang Y, Qu X, Li J, Hale J, Huang Y (2017). Distinct roles for TET family proteins in regulating human erythropoiesis. Blood.

[18] Gascard P, Lee G, Coulombel L, Auffray I, Lum M, Parra M (1998). Characterization of multiple isoforms of protein 4.1R expressed during erythroid terminal differentiation. Blood.

[19] Horne WC, Huang SC, Becker PS, Tang TK, Benz EJ (1993). Tissue-specific alternative splicing of protein 4.1 inserts an exon necessary for formation of the ternary complex with erythrocyte spectrin and F-actin. Blood.

[20] Conboy JG, Shitamoto R, Parra M, Winardi R, Kabra A, Smith J (1991). Hereditary elliptocytosis due to both qualitative and quantitative defects in membrane skeletal protein 4.1. Blood.

[21] Yamamoto ML, Clark TA, Gee SL, Kang J-A, Schweitzer AC, Wickrema A (2009). Alternative pre-mRNA splicing switches modulate gene expression in late erythropoiesis. Blood.

[22] Pimentel H, Parra M, Gee S, Ghanem D, An X, Li J (2014). A dynamic alternative splicing program regulates gene expression during terminal erythropoiesis. Nucleic Acids Res.

[23] Cheng AW, Shi J, Wong P, Luo KL, Trepman P, Wang ET (2014). Muscleblind-like 1 (Mbnl1) regulates pre-mRNA alternative splicing during terminal erythropoiesis. Blood.

[24] Wahl MC, Will CL, Lührmann R (2009). The spliceosome: design principles of a dynamic RNP machine. Cell.

[25] Yoshida K, Sanada M, Shiraishi Y, Nowak D, Nagata Y, Yamamoto R (2011). Frequent pathway mutations of splicing machinery in myelodysplasia. Nature.

[26] Haferlach T, Nagata Y, Grossmann V, Okuno Y, Bacher U, Nagae G (2014). Landscape of genetic lesions in 944 patients with myelodysplastic syndromes. Leukemia.

[27] Malcovati L, Karimi M, Papaemmanuil E, Ambaglio I, Jädersten M, Jansson M (2015). SF3B1 mutation identifies a distinct subset of myelodysplastic syndrome with ring sideroblasts. Blood.

[28] Papaemmanuil E, Cazzola M, Boultwood J, Malcovati L, Vyas P, Bowen D (2011). Somatic *SF3B1* mutation in myelodysplasia with ring sideroblasts. N Engl J Med.

[29] Hu J, Liu J, Xue F, Halverson G, Reid M, Guo A (2013). Isolation and functional characterization of human erythroblasts at distinct stages: implications for understanding of normal and disordered erythropoiesis in vivo. Blood.

[30] Li J, Hale J, Bhagia P, Xue F, Chen L, Jaffray J (2014). Isolation and transcriptome analyses of human erythroid progenitors: BFU-E and CFU-E. Blood American Society of Hematology.

[31] Kim D, Pertea G, Trapnell C, Pimentel H, Kelley R, Salzberg SL (2013). TopHat2: accurate alignment of transcriptomes in the presence of insertions, deletions and gene fusions. Genome Biol.

[32] Trapnell C, Roberts A, Goff L, Pertea G, Kim D, Kelley DR (2012). Differential gene and transcript expression analysis of RNA-seq experiments with TopHat and Cufflinks. Nat Protoc.

[33] Subramanian A, Tamayo P, Mootha VK, Mukherjee S, Ebert BL, Gillette MA (2005). Gene set enrichment analysis: a knowledge-based approach for interpreting genome-wide expression profiles. Proc Natl Acad Sci U S A.

[34] Mootha VK, Lindgren CM, Eriksson K-F, Subramanian A, Sihag S, Lehar J (2003). PGC-1alpha-responsive genes involved in oxidative phosphorylation are coordinately downregulated in human diabetes. Nat Genet.

[35] Vitting-Seerup K, Porse BT, Sandelin A, Waage J (2014). spliceR: an R package for classification of alternative splicing and prediction of coding potential from RNA-seq data. BMC Bioinformatics.

[36] Gautier E-F, Ducamp S, Leduc M, Salnot V, Guillonneau F, Dussiot M (2016). Comprehensive proteomic analysis of human erythropoiesis. Cell Rep.

[37] Enge M, Bao W, Hedström E, Jackson SP, Moumen A, Selivanova G (2009). MDM2-dependent downregulation of p21 and hnRNP K provides a switch between apoptosis and growth arrest induced by pharmacologically activated p53. Cancer Cell.

[38] Vousden KH, Prives C (2009). Blinded by the light: the growing complexity of p53. Cell.

[39] Follis AV, Llambi F, Merritt P, Chipuk JE, Green DR, Kriwacki RW (2015). Pin1-induced proline isomerization in cytosolic p53 mediates BAX activation and apoptosis. Mol Cell.

[40] Chipuk JE, Kuwana T, Bouchier-Hayes L, Droin NM, Newmeyer DD, Schuler M (2004). Direct activation of Bax by p53 mediates mitochondrial membrane permeabilization and apoptosis. Science (80- ).

[41] Spender LC, Carter MJ, O’Brien DI, Clark LJ, Yu J, Michalak EM (2013). Transforming growth factor-β directly induces p53-up-regulated modulator of apoptosis (PUMA) during the rapid induction of apoptosis in myc-driven B-cell lymphomas. J Biol Chem.

[42] Darman RB, Seiler M, Agrawal AA, Lim KH, Peng S, Aird D (2015). Cancer-associated SF3B1 hotspot mutations induce cryptic 3’ splice site selection through use of a different branch point. Cell Rep.

[43] Obeng EA, Chappell RJ, Seiler M, Chen MC, Campagna DR, Schmidt PJ (2016). Physiologic expression of Sf3b1(K700E) causes impaired erythropoiesis, aberrant splicing, and sensitivity to therapeutic spliceosome modulation. Cancer Cell.

[44] Lee E-W, Lee M-S, Camus S, Ghim J, Yang M-R, Oh W (2009). Differential regulation of p53 and p21 by MKRN1 E3 ligase controls cell cycle arrest and apoptosis. EMBO J.

[45] Vassilev LT, Vu BT, Graves B, Carvajal D, Podlaski F, Filipovic Z (2004). In vivo activation of the p53 pathway by small-molecule antagonists of MDM2. Science (80-. ).

[46] Haupt Y, Maya R, Kazaz A, Oren M (1997). Mdm2 promotes the rapid degradation of p53. Nature.

[47] Chen K, Liu J, Heck S, Chasis JA, An X, Mohandas N (2009). Resolving the distinct stages in erythroid differentiation based on dynamic changes in membrane protein expression during erythropoiesis. Proc Natl Acad Sci U S A.

[48] Dussiot M, Maciel TT, Fricot A, Chartier C, Negre O, Veiga J (2014). An activin receptor IIA ligand trap corrects ineffective erythropoiesis in β-thalassemia. Nat Med.

[49] Suragani RNVS, Cadena SM, Cawley SM, Sako D, Mitchell D, Li R (2014). Transforming growth factor-β superfamily ligand trap ACE-536 corrects anemia by promoting late-stage erythropoiesis. Nat Med.

[50] Visconte V, Makishima H, Maciejewski JP, Tiu RV (2012). Emerging roles of the spliceosomal machinery in myelodysplastic syndromes and other hematological disorders. Leukemia.

[51] Hahn CN, Scott HS (2011). Spliceosome mutations in hematopoietic malignancies. Nat Genet.

[52] Dolatshad H, Pellagatti A, Fernandez-Mercado M, Yip BH, Malcovati L, Attwood M (2015). Disruption of SF3B1 results in deregulated expression and splicing of key genes and pathways in myelodysplastic syndrome hematopoietic stem and progenitor cells. Leukemia.

[53] Alsafadi S, Houy A, Battistella A, Popova T, Wassef M, Henry E (2016). Cancer-associated SF3B1 mutations affect alternative splicing by promoting alternative branchpoint usage. Nat Commun.

[54] Wang C, Sashida G, Saraya A, Ishiga R, Koide S, Oshima M (2014). Depletion of Sf3b1 impairs proliferative capacity of hematopoietic stem cells but is not sufficient to induce myelodysplasia. Blood.

[55] Matsunawa M, Yamamoto R, Sanada M, Sato-Otsubo A, Shiozawa Y, Yoshida K (2014). Haploinsufficiency of Sf3b1 leads to compromised stem cell function but not to myelodysplasia. Leukemia.

[56] Matsson H, Davey EJ, Draptchinskaia N, Hamaguchi I, Ooka A, Levéen P (2004). Targeted disruption of the ribosomal protein S19 gene is lethal prior to implantation. Mol Cell Biol.

[57] Khoriaty R, Vasievich MP, Jones M, Everett L, Chase J, Tao J (2014). Absence of a red blood cell phenotype in mice with hematopoietic deficiency of SEC23B. Mol Cell Biol.

[58] Cazzola M, Invernizzi R, Bergamaschi G, Levi S, Corsi B, Travaglino E (2003). Mitochondrial ferritin expression in erythroid cells from patients with sideroblastic anemia. Blood.

[59] Levi S, Corsi B, Bosisio M, Invernizzi R, Volz A, Sanford D (2001). A human mitochondrial ferritin encoded by an intronless gene. J Biol Chem.

[60] Visconte V, Tabarroki A, Zhang L, Parker Y, Hasrouni E, Mahfouz R (2014). Splicing factor 3b subunit 1 (Sf3b1) haploinsufficient mice display features of low risk Myelodysplastic syndromes with ring sideroblasts. J Hematol Oncol.

[61] Danilova N, Sakamoto KM, Lin S (2008). Ribosomal protein S19 deficiency in zebrafish leads to developmental abnormalities and defective erythropoiesis through activation of p53 protein family. Blood.

[62] Jaako P, Flygare J, Olsson K, Quere R, Ehinger M, Henson A (2011). Mice with ribosomal protein S19 deficiency develop bone marrow failure and symptoms like patients with Diamond-Blackfan anemia. Blood.

[63] Dutt S, Narla A, Lin K, Mullally A, Abayasekara N, Megerdichian C (2011). Haploinsufficiency for ribosomal protein genes causes selective activation of p53 in human erythroid progenitor cells. Blood.

[64] Furney SJ, Pedersen M, Gentien D, Dumont AG, Rapinat A, Desjardins L (2013). SF3B1 mutations are associated with alternative splicing in uveal melanoma. Cancer Discov.

