# Supplementary material for: SF3B1 deficiency impairs human erythropoiesis via activation of p53 pathway: implications for understanding of ineffective erythropoiesis in MDS
Source: J Hematol Oncol. 2018 Feb 12;11:19. doi: 10.1186/s13045-018-0558-8 (PMC5810112; doi:10.1186/s13045-018-0558-8)
Supplement: Supplementary file 2 — Expression of SF3B1 during human erythroid differentiation. Figure S2. A schematic structure of each alternative splicing type, along with the associated names and abbreviations. (ZIP 4064 kb) [file 13045_2018_558_MOESM2_ESM.zip › Supplementary Figure legends.docx]

**Supplementary figure legends**

**Supplementary Fig 1. Expression of SF3B1 during human erythroid differentiation. (A)** The mRNA expression levels of SF3B1 as assessed by RNA-seq.**(B)** The mRNA expression levels SF3B1 as assessed by quantitative real-time PCR using β-actin as internal calibrator. **(C)** Western blot analysis of SF3B1. 30 μg of total protein was loaded. **(D)** The protein levels of SF3B1 as assessed by proteomics.

**Supplementary Fig 2.** A schematic structure of each alternative splicing type, along with the associated names and abbreviations.
