# Supplementary figures and images for: SF3B1 deficiency impairs human erythropoiesis via activation of p53 pathway: implications for understanding of ineffective erythropoiesis in MDS
Source: J Hematol Oncol. 2018 Feb 12;11:19. doi: 10.1186/s13045-018-0558-8 (PMC5810112; doi:10.1186/s13045-018-0558-8)

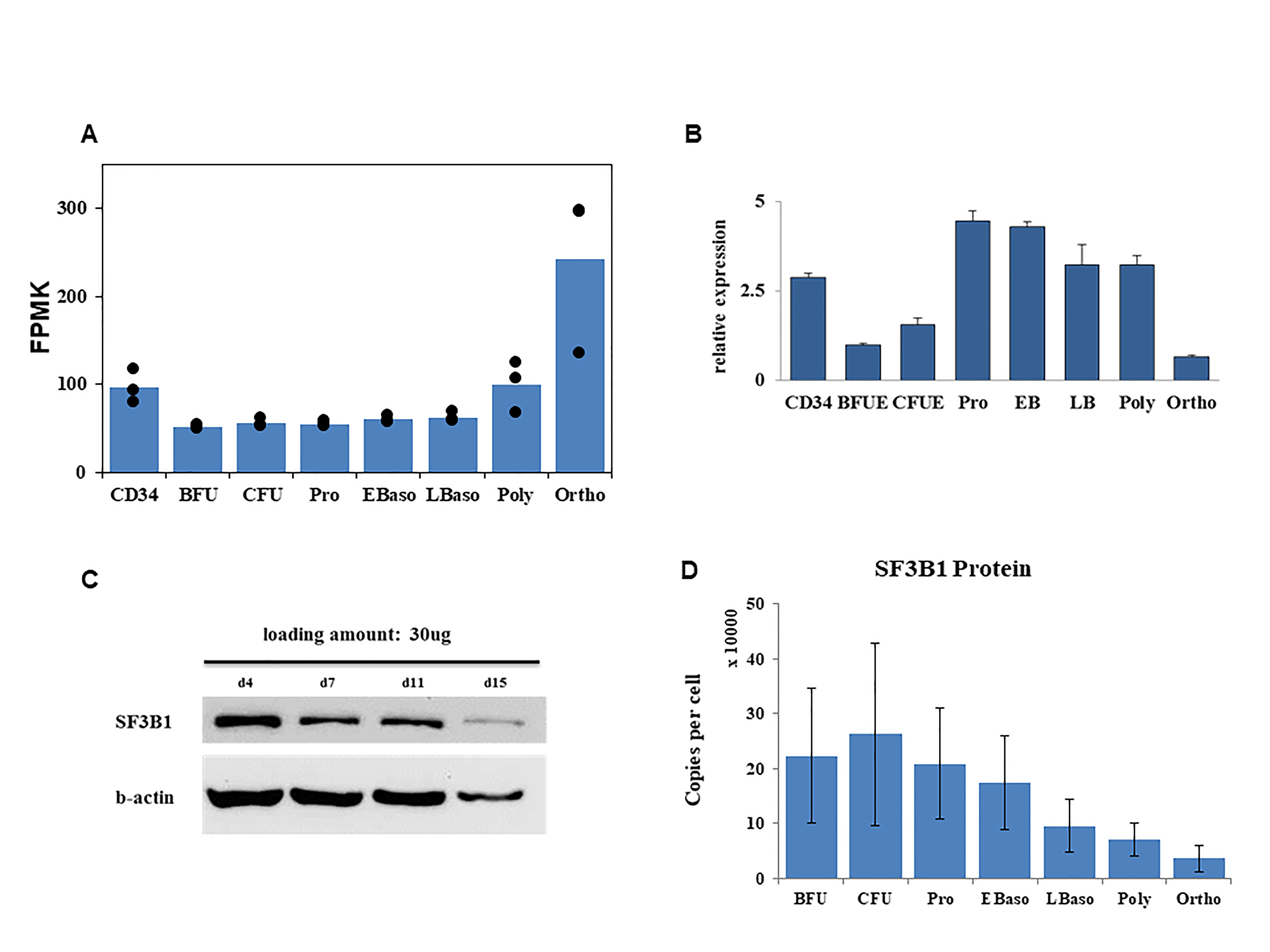

Supplement: Supplementary file 2 — Expression of SF3B1 during human erythroid differentiation. Figure S2. A schematic structure of each alternative splicing type, along with the associated names and abbreviations. (ZIP 4064 kb) [file 13045_2018_558_MOESM2_ESM.zip › Figure S1.tif]

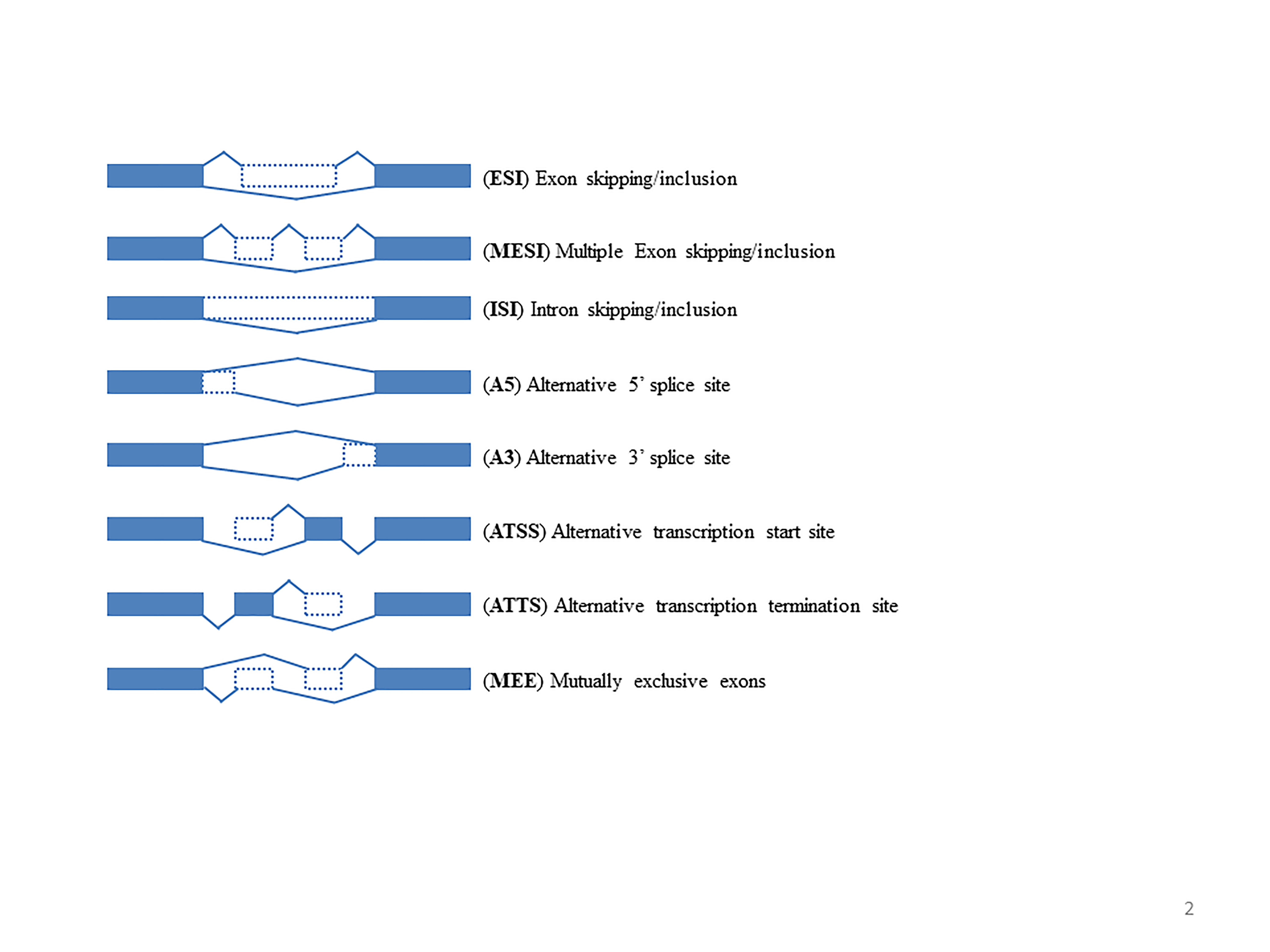

Supplement: Supplementary file 2 — Expression of SF3B1 during human erythroid differentiation. Figure S2. A schematic structure of each alternative splicing type, along with the associated names and abbreviations. (ZIP 4064 kb) [file 13045_2018_558_MOESM2_ESM.zip › Figure S2.tif]
